# A sorghum (*Sorghum bicolor*) mutant with altered carbon isotope ratio

**DOI:** 10.1371/journal.pone.0179567

**Published:** 2017-06-22

**Authors:** Govinda Rizal, Shanta Karki, Vivek Thakur, Samart Wanchana, Hugo Alonso-Cantabrana, Jacque Dionora, John E. Sheehy, Robert Furbank, Susanne von Caemmerer, William Paul Quick

**Affiliations:** 1 C_4_ Rice Center, IRRI, Los Banos, Laguna, the Philippines; 2 Government of Nepal, Ministry of Agricultural Development, Kathmandu, Nepal; 3 International Crops Research Institute for the Semi-Arid Tropics, Hyderabad, India; 4 Australian Research Council Centre of Excellence for Translational Photosynthesis, Division of Plant Sciences, Research School of Biology, The Australian National University, Acton, ACT, Australia; 5 University of Sheffield, Sheffield, United Kingdom; Universidad Miguel Hernández de Elche, SPAIN

## Abstract

Recent efforts to engineer C_4_ photosynthetic traits into C_3_ plants such as rice demand an understanding of the genetic elements that enable C_4_ plants to outperform C_3_ plants. As a part of the C_4_ Rice Consortium’s efforts to identify genes needed to support C_4_ photosynthesis, EMS mutagenized sorghum populations were generated and screened to identify genes that cause a loss of C_4_ function. Stable carbon isotope ratio (*δ*^13^C) of leaf dry matter has been used to distinguishspecies with C_3_ and C_4_ photosynthetic pathways. Here, we report the identification of a sorghum (*Sorghum bicolor*) mutant with a low δ^13^C characteristic. A mutant (named Mut33) with a pale phenotype and stunted growth was identified from an EMS treated sorghum M_2_ population. The stable carbon isotope analysis of the mutants showed a decrease of ^13^C uptake capacity. The noise of random mutation was reduced by crossing the mutant and its wildtype (WT). The back-cross (BC_1_F_1_) progenies were like the WT parent in terms of ^13^C values and plant phenotypes. All the BC_1_F_2_ plants with low δ^13^C died before they produced their 6^th^ leaf. Gas exchange measurements of the low δ^13^C sorghum mutants showed a higher CO_2_ compensation point (25.24 μmol CO_2_.mol^-1^air) and the maximum rate of photosynthesis was less than 5μmol.m^-2^.s^-1^. To identify the genetic determinant of this trait, four DNA pools were isolated; two each from normal and low δ^13^C BC_1_F_2_ mutant plants. These were sequenced using an Illumina platform. Comparison of allele frequency of the single nucleotide polymorphisms (SNPs) between the pools with contrasting phenotype showed that a locus in Chromosome 10 between 57,941,104 and 59,985,708 bps had an allele frequency of 1. There were 211 mutations and 37 genes in the locus, out of which mutations in 9 genes showed non-synonymous changes. This finding is expected to contribute to future research on the identification of the causal factor differentiating C_4_ from C_3_ species that can be used in the transformation of C_3_ to C_4_ plants.

## Introduction

Stable carbon isotope ratios (*δ*^13^C) and carbon isotope discrimination (Δ^13^C) are used to distinguish C_4_ and C_3_ plants. Discrimination against ^13^C during carbon fixation is greatly dependent on the photosynthetic type, mainly due to the characteristics of the enzyme catalyzing the first step in carbon fixation. Carbon isotope discrimination is defined as Δ = R_air_/R_p_-1 where R_air_ and R_p_ stand for the ^13^C/^12^C ratio in the air and the photosynthetic product, respectively [1,2]. In C_3_ plants, this step is mediated by Rubisco, which has a strong preference for CO_2_ containing the lighter isotope ^12^C over the heavier and less abundant ^13^C. In C_4_ plants, inorganic carbon is initially fixed by Phospho*enol*pyruvate carboxylase (PEPC) that does not discriminate between the two isotopes. The C_4_ acid is then transferred to the bundle sheath cell where it is decarboxylated and the CO_2_ accumulates to high concentrations. Leakages of CO_2_ from the bundle sheath cells to the mesophyll cells are prevented by increased diffusive resistance of the bundle sheath cell walls,as such Rubisco is given no option but to fix both isotopes of carbon and hence the isotope discrimination is much lower in C_4_ than in C_3_ plant materials. As a result, the two photosynthetic types can be clearly distinguished by their signatures in carbon isotopic discrimination [3,4]. Interestingly, incomplete C_4_ photosynthesis in intermediate C_3_-C_4_ species can also be detected by its effect on Δ^13^C [5,6]. Differential diffusion of ^13^CO_2_ and ^12^CO_2_ through stomata is another major component of overall discrimination [7]. In an ideal atmospheric condition, the ratio of ^13^C to ^12^C is roughly 1:99 [8]. The ratio of ^13^C /^12^C in the plant dry matter reflects the photosynthetic discrimination that occurred during its lifetime [9]. These variations in isotope ratios are integrated into the isotopic signature of leaf dry matter (*δ*^13^C) [3,10]which is usually referenced to the standard Pee Dee Belemnite (PDB) and defined as δ = R_p_/R_PDP_−1, where R_p_ and R_PDB_stand for the ^13^C/^12^ Cratio in leaf dry matter and the standard PDB, respectively [8]. The measurements of stable carbon isotope ratios (*δ*^13^C) and carbon isotope discrimination (Δ^13^C) are used to distinguish the photosynthetic efficiency of plants [3]. C_3_ plants have *δ*^13^C values between -23 and -35*‰* which is lower than the *δ*^13^C values of C_4_ plants which are between-10 and -14*‰* [10]. The genetic factors responsible for the differences in δ^13^C between C_3_ and C_4_ plants have been the interest of scientists for a long time.In maize, one of the model C_4_ species, the Δ^13^C was proposed to be under polygenic control[11]. We hypothesized that induced mutations can revert a C_4_ plant to a C_3_ or an intermediate type and this reversion can be detected from its δ^13^C signatures. The seeds of one of the accessions of the first sequenced sorghum (BTx623-Rooney) [12] were mutagenized using EMS and mutant seedlings screened to find those with altered δ^13^C values. Several mutants, showing reduced (more C_3_-like) δ^13^C were isolated for whole genome sequencing (WGS) [13] to map underlying mutations. The sequence analysis and the comparison between WT and mutants lead to the identification of several structural variances [14]. We were able to identify a potential genetic region controlling carbon isotope discrimination. These findings are useful for the identification of genetic factors driving the evolution of the C_4_ photosynthetic pathway.

## Results

### Identification of sorghum mutant with low *δ*^13^C values

**Fig 1:**
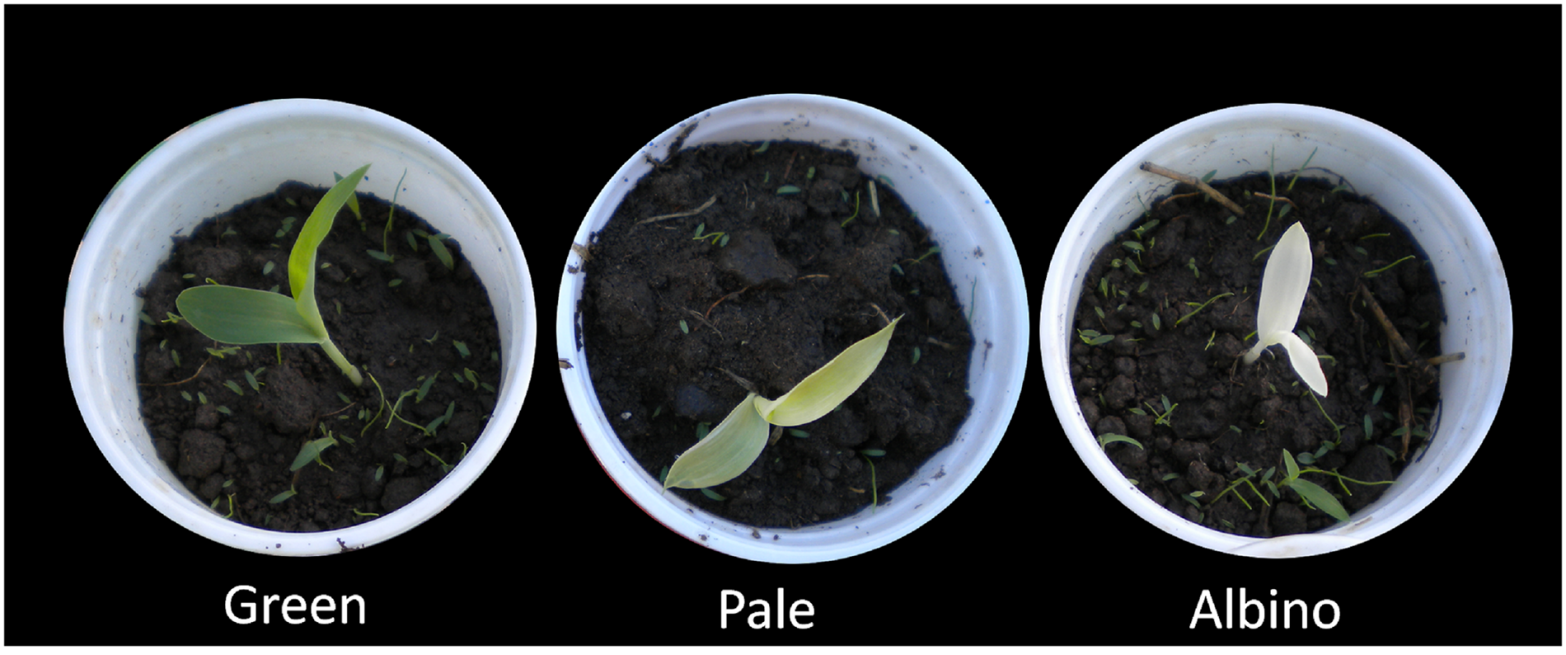
Representative images of notable phenotypes observed in EMS treated sorghum M_1_ population. The percentage of green, pale and albino were 92, 4, and 4, respectively in the M_1_ population.

**Fig 2:**
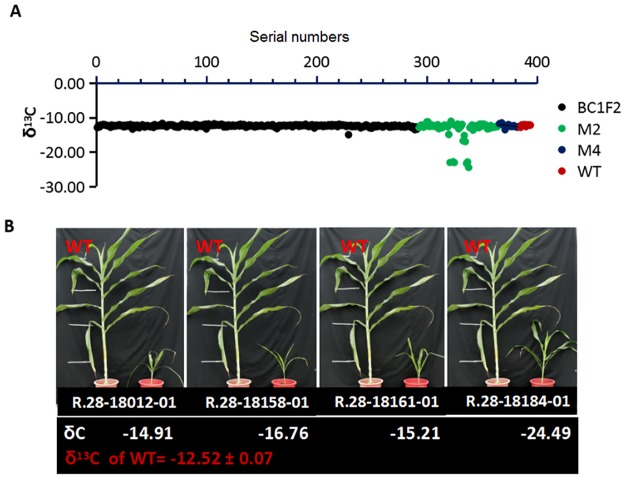
The carbon isotope ratio of the EMS mutants of different generations and wildtypes (WT). (A) The δ^13^C values of selected wildtype and mutant sorghum plants accessions. The data of two generations of mutants M_2_ and M_4_ are shown. At M_2_ generation the mutant was identified and the surviving M_4_ corresponds to BC_1_F_2_ generation. The red dots represent WT values, the black dots represent mutants with normal δ^13^C, and the green dots represent the plants selected for further analysis. (B) A comparison of phenotypes and δ^13^C values between low δ^13^C mutants and WT. For convenience, the names of four mutants R.28-18184-01, R.28-18158-01, R.28-18161, and R.28-18012-01 were renamed as Mut31, Mut32, Mut33, and Mut34, respectively.

The M_1_ seedlings had green (92%), pale (4%) and albino (4%) phenotypes (Fig 1). From a population of one million M_1_ seeds, 35,000 individual panicles were advanced to the M_2_ generation. A total of 66 EMS mutants were selected based on their deviation from WT phenotypes and the δ^13^C values of leaf dry matter of the mutants were measured. Four EMS mutants had a lower δ^13^C than the WT (Fig 2). The four mutants,R.28-18184-01, R.28-18158-01, R.28-18161, and R.28-18012-01 with δ ^13^C values of -24.49, -16.76, -15.21 and -14.91were labeled as Mut31, Mut32, Mut33, and Mut34, respectively (Fig 2). The δ^13^C values of WTs grown in the same environment ranged between -12.89 and -12.55 (Fig 2). The M_2_ progenies of the mutants were smaller, paler and slower growing than the WT (Fig 2B). However, only the Mut33 produced seeds.

### Generation of Mut33 backcrossed population for sequencing

Mut33 was crossed to its WT and part of the panicle was self-pollinated to generate M_3_ seeds. Six back-crossed F_1_ (BC_1_F_1_)seeds from the cross between Mut33 and WT-Rooney and 18 self pollinated (M_3_) seeds were harvested. The BC_1_F_1_ plants grew normally and produced seeds at the same time as the WT. More than 3,000 seeds were harvested from a single plant. Both low δ^13^C and normal δ^13^C seedlings were identified in BC_1_F_2_ population. The M_3_ seeds did not germinate in soil. Some M_3_ seeds germinated in plant growth medium, but they could not survive beyond the sixth leaf stage. They were pale and the leaves desiccated starting from the tip of the leaves and died, even when grown under elevated CO_2_ (10000ppm), controlled temperature, low light, and long day conditions. Even then they died before maturity. The BC_1_F_2_ seedlings were used for further analysis.

### Characterization of BC_1_F_2_ progenies of Mut33

The δ^13^C values in M_2_, M_3_, BC_1_F_1_ and BC_1_F_2_ population obtained from Mut33 were-15.21 ‰, -13.79 ‰ ± 0.19, -12.95 ‰ and -13.22 ‰ ± 0.72, respectively. The average (±SD) WT δ^13^C values across the generations was -12.98 ‰ ± 0.13. The BC_1_F_1_ had WT-like δ^13^C values and phenotypes. In BC_1_F_2_ the range of δ^13^C values broadened as indicated by large standard deviation (0.72) and ranged between (-15.61 and -12.06 ‰), showing a clear segregation of the δ^13^C trait in the population. The samples were pooled according to their δ^13^C values: SbPool1 (-14.319 to -13.5869), SbPool2 (-13.5762 to -13.3126), SbPool3 (-12.7924 to -11.1609) and SbPool4 (-12.6585 to -12.2538) (Table 1). Each pool had 30 samples and there were two pools from the first type of seedling. There were 115 seedlings with low δ^13^C that died before producing sixth leaves, 31 seedlings with normal δ^13^C which were slow growing and 51 normal seedlings with normal δ^13^C values.

**Table 1:** Whole genome sequencing of pooled samples.

Sample	δ^13^C values	Phenotypic traits	No. of paired-end reads (Millions)	Read length (bp)	No. of base pairs (Gbp)	Sorghum genome coverage
SbPool 1	-14.319 to -13.5869	Small plants, lethal	93.467	125	23.37	32X
SbPool 2	-13.5762 to -13.3126	Small plants, lethal	98.724	125	24.68	34X
SbPool 3	-12.7924 to -11.1609	Small plants, survived	135.059	125	33.76	46X
SbPool 4	-12.6585 to -12.2538	Tall plants, survived	129.253	125	32.31	44X

### Chlorophyll content

**Fig 3:**
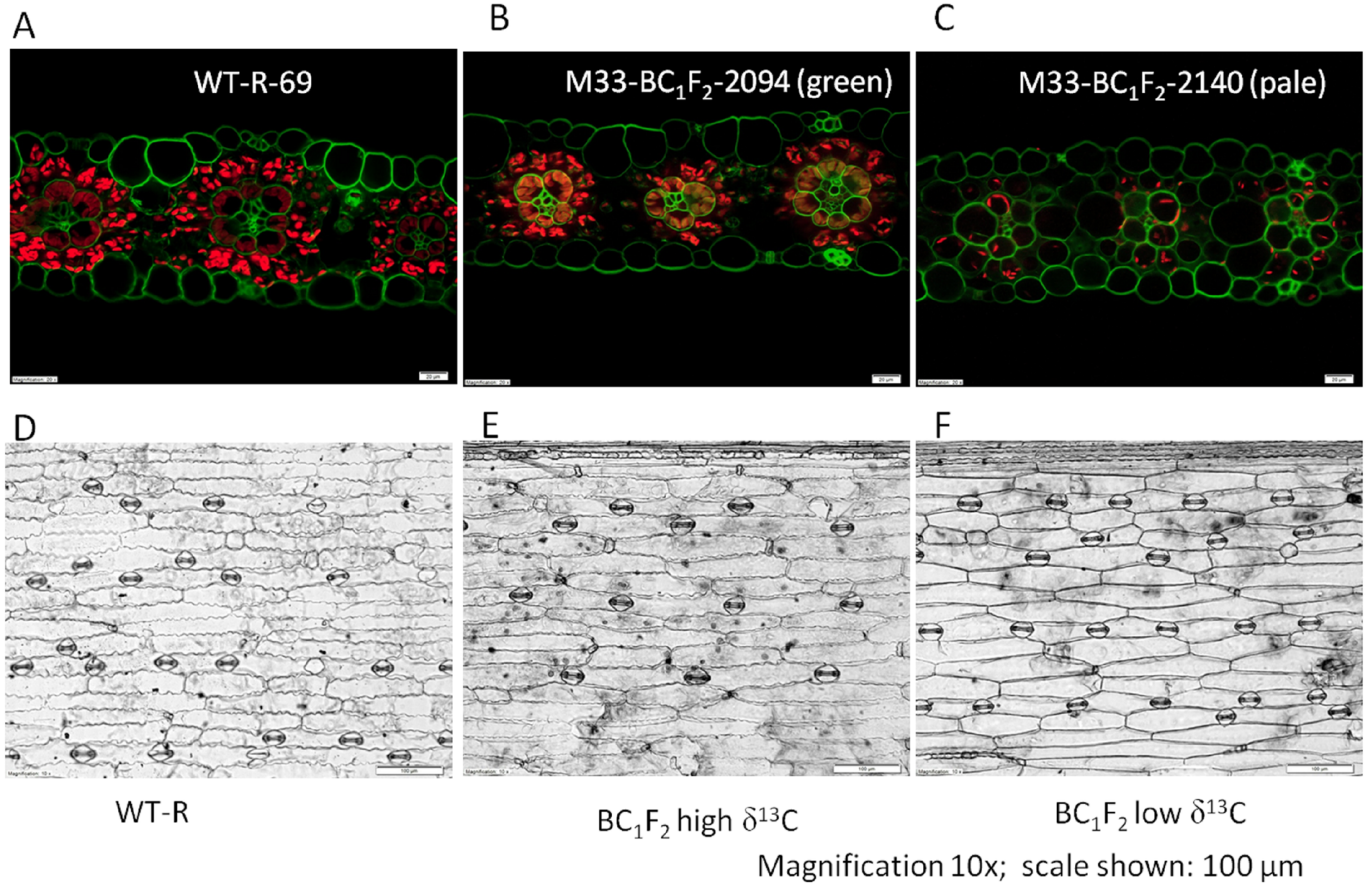
Free hand sections of fresh leaves of (A) WT, (B) normal δ^13^C BC_1_F_2_ and (C) low δ^13^C BC_1_F_2_ seedlings. The red dots show the presence of chlorophyll in the leaf section. (D,E, and F) The stomatal densities of the seedlings are in the same order as above. The low δ^13^C BC_1_F_2_ mutants had more stomata per mm^2^ than the WT/normal. Scale: in A, B, and C the magnification is 20x and the scale shown is 20 μm; in D, E and F magnification is 10x and the scale shown is 100 μm.

A strong positive correlation was found between δ^13^C and greenness in BC_1_F_2_ plants(r = 0.77; n = 197; using STAR Pearson’s correlation analysis). This was explained by lower chlorophyll content in low δ^13^C mutants. In free hand sections of the fresh leaves observed under a high-resolution microscope, the number of chloroplasts, seen as fluorescent red dots, was lower in the low δ^13^C mutant plants. In the WT, the bundle sheath cells were full of chloroplasts and many chloroplasts were seen in mesophyll cells. In the BC_1_F_2_ plants, with normal δ^13^C, the number and distribution of chloroplasts were comparable to the WT. The chloroplasts number in the low δ^13^C mutants had decreased substantially both in bundle sheath and mesophyll cells (Fig 3A–3C). The reduction in the number of chloroplasts in the BS cells as well as total number of chloroplasts in low δ^13^C mutants, which correlated with reduced photosynthesis, could be the cause of early death in the mutants, as the absence of chloroplasts correlated with the significantly reduced rate of photosynthesis.

#### Stomatal density

The mutants with low δ^13^C in BC_1_F_2_ had significantly higher stomatal density than the mutants with normal δ^13^C and the WT (P ≤0.05). The average density of leaf stomata in low δ^13^C, normal δ^13^C, and WT were 122 ± 0.52, 60± 0.50 and 72± 2.45 stomata per mm^2^, respectively (Fig 3B). The stomatal density was not significantly different between the normal δ^13^C and the WT.

### Gas exchange measurement

**Fig 4:**
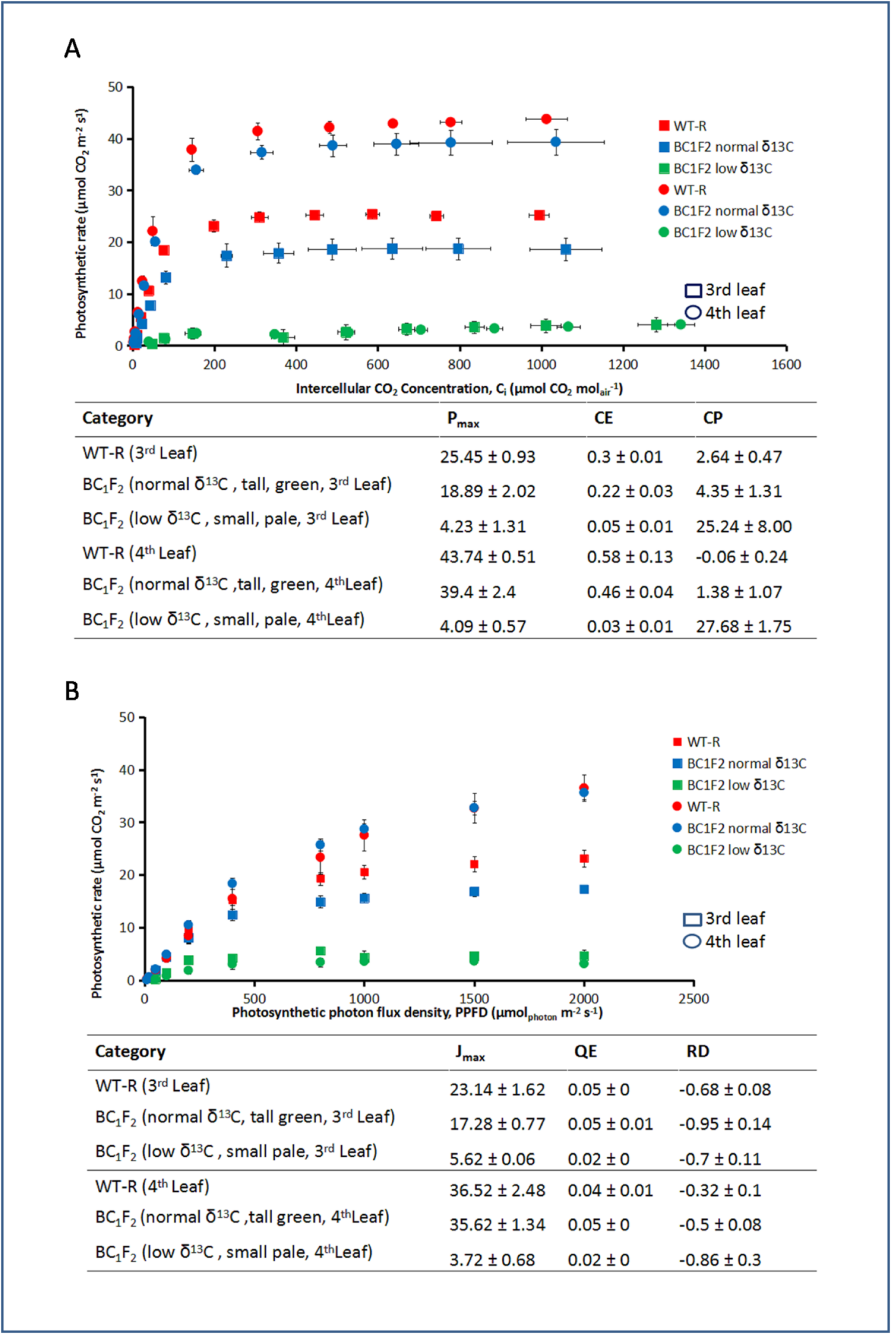
Measurement of photosynthetic parameters of BC_1_F_2_ mutants and wild type. (A)The photosynthesis rate per internal CO_2_ concentration response curves (ACi) for the low δ^13^CBC_1_F_2_ (green square), normal δ^13^CBC_1_F_2_ (blue square) and wildtype (WT) (red square). The maximum rate of photosynthesis (P_max_), carboxylation efficiency (CE) and CO_2_ compensation points (CP) are shown in the table. However, the CO_2_ compensation point is high for low δ^13^C mutants. The squares show data of 3^rd^ leaf and circles for the 4^th^ leaf. The green, blue, and red colors represent low δ^13^C BC_1_F_2_, normal δ^13^C BC_1_F_2_, and WT, respectively. (B)The light response curve (LRC) for the low δ^13^C BC_1_F_2_ (green square) normal δ^13^CBC_1_F_2_ (blue square) and wildtype (red square). The maximum rate of photosynthesis (J_max_), quantum efficiency (QE) and light compensation point (RD) are shown in the table. The squares show data of 3^rd^ leaf and circles of the 4^th^ leaf. The green, blue and red marks represent low δ^13^C BC_1_F_2_, normal δ^13^C BC_1_F_2_, and WT, respectively.

We assumedthat the first three leaves of a seedling are sustained by the nutrients stored in the seed and from the fourth leaf onward the photosynthesis supports complete autotropism. We observed the fourth leaf as the transition point for lethality in the BC_1_F_2_ plants with low δ^13^C. Thus, we took gas exchange measurements on the 3^rd^ and 4^th^ leaves. The A/C_i_ curves showed the P_max_ for the low δ^13^C BC_1_F_2_ (green), normal δ^13^C BC_1_F_2_ (blue) and WT (red) were 4.23, 18.89 and 25.45 μmolCO_2_ m^-2^s^-1^ for the third leaf (square marker) and 4.09, 39.4, 43.74 μmolCO_2_ m^-2^s^-1^ for the fourth leaf (circle marker), respectively (Fig 4A). The CO_2_ compensation point (CP) had increased to 25.24 and 27.68 μmolCO_2_mol_air_^-1^ for the third and the fourth leaf of low δ^13^C mutants, respectively. The CP for the normal δ^13^C BC_1_F_2_ and WT (Fig 4A) was within the range of C_4_ species (0 to 12 ppm) [15].

The J_max_ calculated from the light response curve for the low δ^13^C BC_1_F_2_ (green), normal δ^13^C BC_1_F_2_ (blue) and WT (red) were 5.62, 17.28, and 23.14 μmolCO_2_m^-2^s^-1^ for the third leaf (square) and 3.72, 35.62, and 36.52 μmolCO_2_ m^-2^s^-1^ for the fourth leaf (circle), respectively (Fig 4B). There were no significant alterations in quantum efficiency and light compensation points.

One fourth of the BC_1_F_2_ seedlings died before the sixth leaf stage. Those lethal plants had a high CP and low P_max_.

### Whole genome sequencing and discovery of candidate genes

Four pools of DNA samples were sequenced at 32–46 X coverage (Table 1). The overall quality of sequence reads was good as filtering/trimming of low-quality reads or bases lost less than one percent of reads (Table 2). Since the reads were filtered and aligned against the WT parental genome (i.e., BTx623-Rooney), a very high percent of reads (~95%) aligned successfully to the reference genome (Table 2). Variants were discovered jointly in four samples followed by their filtering using multiple criteria (see Methods). The number of EMS-induced SNPs in the four samples ranged between 22,000 and 25,000 (S1 Fig).

**Table 2:** Sequence data analysis and variant discovery.

Sample	Raw unpaired reads (in Millions)	Unpaired reads passed filtering (in millions)	% passed	No. of clean reads (in millions)	No. of mapped reads (in millions)	Percent alignment	No. of variants
SbPool 1	186.93	185.69	99.33	185.69	176.68	95.14	10694
SbPool 2	197.44	196.16	99.34	196.16	189.74	96.72	10910
SbPool 3	270.11	267.65	99.08	267.65	247.54	92.48	10694
SbPool 4	258.5	256.15	99.09	256.15	252.04	98.39	10694

### Identification of causal locus

**Fig 5:**
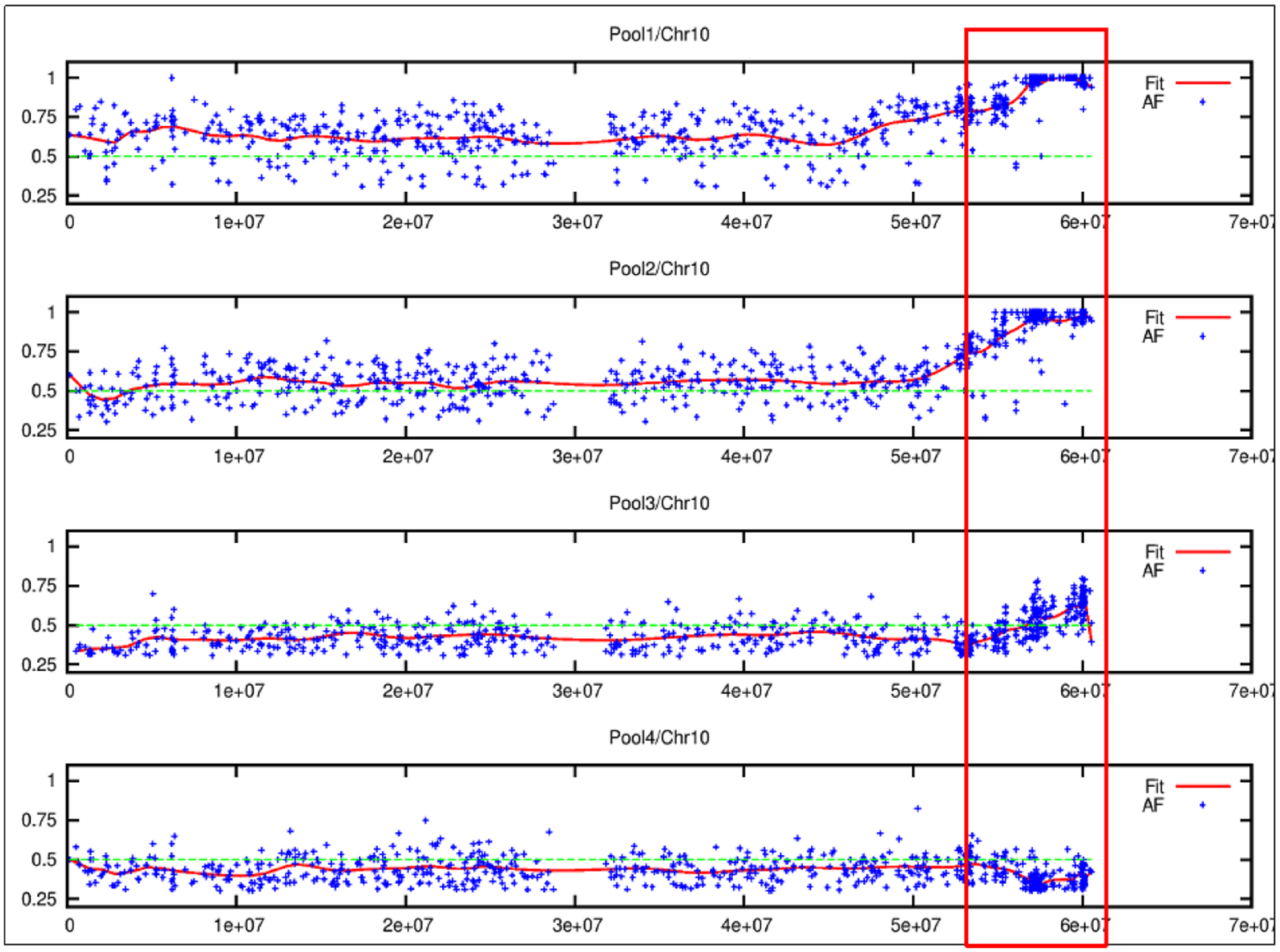
The focused image of q arm of chromosome 10 where the alternate allele frequency was high (≈1) in SbPool 1 and SbPool 2, the pools with low δ^13^C and fatal samples about 0.5 in SbPool 3, the pool with normal δ^13^C but slow growing samples and about 0.33 in SbPool 4, the pool with normal δ^13^C and phenotype.

To identify the region of the genome associated with the mutant phenotype using a bulk segregant strategy, the frequency of mutant/alternate alleles (AF) was plotted along the genome [15]for all pooled samples. The density of AF was uniform across all chromosomes except at the end of the tenth chromosome (S2 Fig). Clearly, the end of the q-arm of chromosome 10 had a region where the mutant allele frequency in mutant pool (SbPool 1) rose to 1 and then declined (Fig 5, or S2 Fig), whereas in non-mutant pool (SbPool 4) the allele frequency in the same region was much lower than the average value of 0.5, which agreed with the expected allele frequency in the non-mutant pool of 0.33. The SNPs were extracted from this region of chromosome 10 (S2 Fig) and were annotated to identify those that can potentially affect the protein sequence. Out of 211 SNPs, ~70 SNPs overlapped with the gene boundaries (including core promoters and/or UTRs but excluding introns); only 9 of them caused a change in the amino acids (S3 Table). A database search for information on the function of these genes revealed that only four had curated information available: two of them (Sobic.010G239700 and Sobic.010G241900) have protein kinase activity, the third one (Sobic.010G249000) was a disease resistance gene, while the fourth one (Sobic.010G250100) showed homology with a transcription factor involved in chloroplast targeted protein import (Table 3). Among the uncharacterized genes, the cellular location of one of the genes (Sobic.010G266000) was predictedto be related to the plastid and its ortholog in maize shows abundance exclusively in leaf tissues (S3 Fig). There are two candidates that show a very clear association with the chloroplast. A transcription factor (TF) predicted to be involved in chloroplastic protein import (Sobic.010G250100) [16] was found to have very high transcript abundance in the maize primordial tissues and almost nil in the foliar immature and expanded stages (S4 Fig). Expression profile of all the candidates showed a variation in expression across different tissues (S5 Fig).

**Table 3:** The SNPs, from the locus of the genome associated with the mutant phenotype, causing amino acid changes.

SN	Gene ID	Position (BTx623)	Amino acid changes	Annotation
1	Sobic.010G239700	57965771	Ala>Thr	cysteine-rich RLK (RECEPTOR-like protein kinase) 10
2	Sobic.010G239900	57978990	Cys->Tyr	2-oxoglutarate (2OG) and Fe(II)-dependent oxygenase superfamily protein
3	Sobic.010G241900	58127862	Leu>Phe	Leucine-rich receptor-like protein kinase family protein
4	Sobic.010G249000	58665427	Asp>Asn	NB-ARC domain containing
5	Sobic.010G250100	58730675	Pro>Leu	CIL, CIA2 like (Chloroplast import apparatus)
6	Sobic.010G263800	59789763	Gly>Asp	uncharacterized protein
7	Sobic.010G266000	59985708	Pro>Ser	uncharacterized protein
8	Sobic.010G264000	59800420	Val>Ile	expressed protein
9	Sobic.010G265600	59962313	Val>Ile	Protein of unknown function (DUF581)

### Gene expression analysis of the major candidate genes

**Fig 6:**
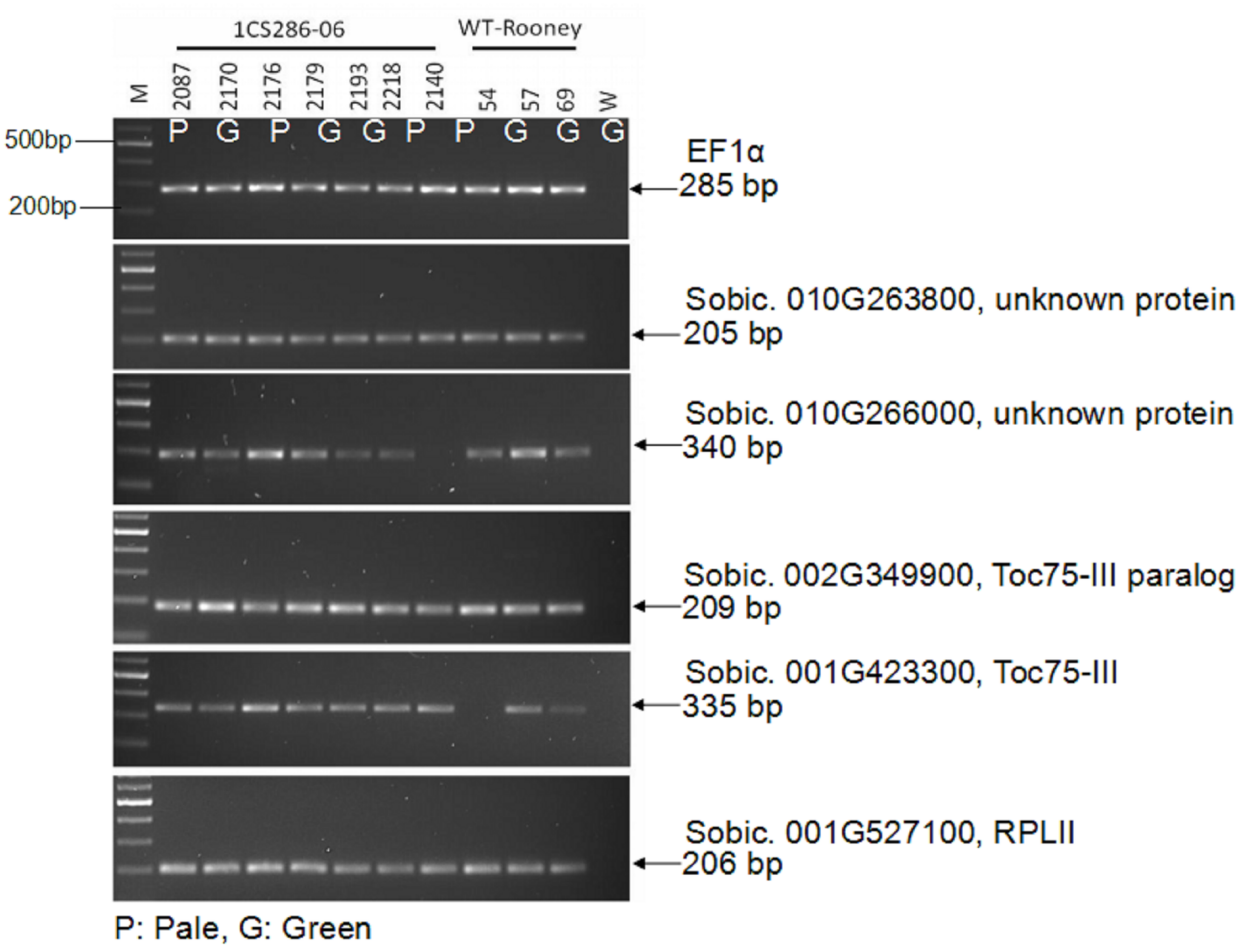
Semi-quantitative PCR analysis of candidate genes of Mut33 BC_1_F_2_ seedlings. The bands represent expression of Sobic.010G263800, Sobic.010G266000, and the three downstream genes of CIA2, Sobic.002G349900 (TOC75-III paralog), Sobic.001G423300 (TOC75-III), and Sobic.001G527100 (RPL11) in BC_1_F_2_ (1CS286-06) and wildtype (WT-Rooney) samples. There are no changes in gene expression levels of TOC75-III, and RPL11. EF1α is the housekeeping gene.

Candidate genes were tested for transcript expression using RNA from the third leaf stage. Results from semi-quantitative PCR showed that transcripts of Sobic 010G263800 and Sobic 010G266000, both annotated as uncharacterized proteins were amplified at similar levels in WT and the pale mutants (Fig 6). Sobic 010G239700 showed faint amplification in the two samples while Sobic 010G24900 could not be amplified (S6 Fig). The most promising candidate gene namely CIA2 like (chloroplast import apparatus) Sobic 010G250100 showed faint amplification only in three samples, which could be due to the RNA from the leaf tissue as this gene was found to express in the primordial stage in maize(Fig 6). Therefore, three CIA2 pathway genes Toc75-III (Sobic 001G423300), Toc75-III paralog (Sobic 002G349900) and RPL11 (Sobic 001G527100) were analyzed. All three showed similar levels of transcript abundance in the mutants and WT (Fig 6, S6 Fig). Other genes in the CIA2 pathway that are related to chloroplast formation and development could be tested to gain more insight of the causal gene.

## Discussion

The mutants were first selected through visual inspection of deviation from WT phenotypes. These mutants were slower growing and paler compared to the WT, similar characteristics were observed in mutants with defective CIA2, one of the transcription factors involved in chloroplast protein import [17]. The sorghum mutants had high mortality and low δ^13^C. The differences in degree of stable carbon isotope discrimination between C_3_ and C_4_ plants can be used to screen mutants with the loss of function of the C_4_ pathway. In maize, another C_4_ species, the Δ^13^C was demonstrated to be under polygenic control [11]. There are no reports on the genetic factors controlling ^13^C composition in sorghum. An inverse relationship between grain yield and carbon isotope discrimination probably due either to the porosity of BS cells affecting the light use efficiency or the differences in CO_2_ assimilation rate and stomatal conductance due to variable transpiration efficiency, was previously reported [18]. The sorghum mutants identified in this study had a higher stomatal density (Fig 3F), fewer chloroplasts (Fig 3C), lower carboxylation efficiency and higher CO_2_ compensation point compared to the WT (Fig 5A). The range and average δ^13^C are -23 to -35 ‰ and -26 ‰ in C_3_ plants and from -10 to -14 ‰ and -13 ‰ in C_4_ plants, respectively [10]. We found that even small changes in carbon isotope ratios were correlated with lethal effects in the plants. Is the lethality in the mutants controlled by the same factors controlling the ^13^C composition or some major genes directly or pleiotropically affecting the ^13^C composition?

The CO_2_ response curve indicated that the photosynthetic capacities of these mutants were impaired compared to the WT. The phenotype was recovered in the BC_1_F_1_ generation. The photosynthetic impairment was severe to lethal in one-fourth of the seedlings in the BC_1_F_2_ generation. Unlike in maize [19] where ^13^C amount is controlled by polygenes, the alteration of δ^13^C in sorghum mutants is either a single recessive gene or mutation in a vital gene or in a pathway that affected the assimilation of ^13^Cand caused premature senescence. The available mutant populations are important materials to study the genetic factors controlling the indiscriminate uptake of ^13^C isotope by C_4_ plants, which is absent in C_3_ plants The alignment of DNA sequences from BC_1_F_2_ mutants with low and normal δ^13^C against the WT genome sequence showed a consistently high alternate frequency (AF) in the tail (q) end of chromosome 10. Based on the consistently high AF, we propose the factor responsible for δ^13^C variation between WT and the mutants is in the locus between the 57,941,104 and 59,985,708 bp. Mutations affecting 37 genes were found in that locus (S2 Table) of which mutations in 9 genes had caused non-synonymous changes (Table 3). The functional annotation of Sobic.010G239700 was cysteine-rich repeat receptor-like protein kinase (*CRR-RLK*) [20–23]. The *CRR-RLKs* are receptor-like kinases in plants with roles in signal transduction in response to extracellular stimuli and stimulation of downstream pathways. The *CRR-RLKs* are involved in growth regulation, development, and physiological responses [24]. In *Arabidopsis*, they are activated in response to UV rays and pathogen-response [24, 25]. The Sobic.010G239900 has roles in oxidoreductase activity [20, 21]. Oxido-reductase activity uses ferrous iron as a cofactor to catalyze 2-oxoglutarate into succinate [20]. The Fe (II) 2OG dioxygenase domain enzymes in plants catalyze the production of plant hormones, such as ethylene, gibberellins, anthocyanidins and pigments such as flavones [26]. Sobic.010G239900is one of the Leucine-rich repeat receptor-like protein kinases (*LRR-RLK*) [27]. The *LRR-RLKs* are transmembrane receptor-like kinases in plants. They regulate various developmental and defense-related processes such as cell proliferation, stem cell maintenance, and hormone perception [28–30]. They play an important role in pathways of brassinosteroid signaling in the wound-responsive signaling pathway in Solanaceous plants, nodule development in leguminous plants and pathogen-recognition in *Arabidopsis*[28, 30]. The *LRR-RLKs* are an integral component of membranes consisting of gene products and complexes having peptides embedded in the hydrophobic region of the plasma membrane. They perform transmembrane signaling receptor activity that transmits a signal across the membrane via kinase activity or phosphorylation of amino acid residues in a protein such as threonine and serine signaling a wound-response [20]. The Sobic.010G241900 has a functional annotation of Leucine-rich repeat-containing protein [27, 31]. The gene has roles in defense responses to foreign bodies or injuries to reduce damage [27, 31, 32]. The geneSobic.010G250100 is similar to putative stress resistance-related protein [30]. One of the proteins is related to CHLOROPLAST IMPORT APPARATUS 2 (CIA2) e.g. At5g57180 [33]. The mutation of this gene results in a pale phenotype that is defective in the general chloroplast protein import pathway. Its subcellular location is in the chloroplast and nucleus and is expressed in young leaves and flower buds [33]. The gene CIA2 is crucial for development of photosynthetic apparatus but it is not known if it also has a pleiotropic effect on the carbon concentrating mechanism. Sobic.010G250100 is responsible for specific up-regulation of the translocon genes TOC33 and TOC75 in leaves. It is also involved in the general chloroplast protein import pathway regulation, including protein import and protein translation efficiency [33, 34]. The biological process includes protein targeting to chloroplasts [33] and regulation of transcription [34]. The gene Sobic.010G263800 encodes a protein that belongs to uncharacterized protein family (UPF0183 protein) [20, 23, 27, 31]. The gene is involved in the response to symbiotic fungus [35]. The Sobic.010G266000 (GRMZM2G373420) has no functional annotation (http://phytozome.jgi.doe.gov/pz/portal.html). The gene Sobic.010G264000 (GRMZM2G015818) encodes a protein of unknown function (DUF1668). The hypothetical proteins found in this family are expressed in *Oryza sativa* and are of unknown function [36]. The gene Sobic.010G265600 (GRMZM2G009080) or “DUF581” is a Zf-FCS type zinc finger. Zinc fingers are a ubiquitous class of protein domains with considerable variation in structure and function. FCS-like zinc finger proteins have small motifs with multiple finger-like protrusions that make tandem contacts with their target molecule with which they interact. Some of its functions are (1) modular building blocks for the construction of larger protein domains that recognize and bind to specific DNA sequences [37], (2) DNA-binding transcription factors [17] and (3) recognition of RNA and other protein [38]. Transcript abundance of the eight candidate genes were examined which showed only marginal differences. Finer analysis and study of the effect of individual genes could reveal the genetic factors responsible for the differentiation of δ^13^C in C_3_ and C_4_ plants. The causal gene is most likely to be one of the genes mentioned above (Table 3) unless the causal mutation lie outside the coding sequence of a gene, or in a sequence not annotated as a gene (such as a micro RNA sequence or an incorrectly annotated gene). But given the mutant phenotype characterized by significantly reduced number of chloroplasts, and one of the candidate genes being a transcription factor annotated to regulate chloroplast protein import during chloroplast development (CIA2 like), and this being transcriptionally active in the developmental stages of leaf known for plastid biogenesis and differentiation, we propose this gene to be most likely causal gene.

If carbon comes from the seed then it will have the C_4_ signature of the parent which was not the case. If carbon comes from recently fixed CO_2_, it reflects the type of pathway (C_3_ or C_4_) which was more C_3_ like in the mutants. Therefore, we assume that something has gone wrong in the mutant with the C_4_ pathway. It is the current photosynthetic pathway that is providing the sugar for growth with a more C_3_-like signature. Moreover, the screen based on ^13^C was effective for the identification of photosynthetic mutants and was a useful parameter to cross-check successful crossings.

In conclusion, our assumption is that the C_4_ pathway has been compromised and the cost of CO_2_ concentration has been increased leading to a negative carbon balance and hence is lethal. So, whilst development of C_4_ may be the cause, the end result is loss of C_4_ function. Further experiments are needed to pin down the causal trait to the gene level. This finding is expected to help future research on the genetic control of carbon isotope discrimination in plants.

## Methods

### Plant materials

Sorghum (*Sorghum bicolor* L. [Moench]) BTx623 seeds were obtained from Professor William L. Rooney of the Sorghum Breeding and Genetics Division (Texas A & M University, College Station, Texas, USA). Hence, the accession was named BTx623-Rooney or WT-R [12]. Cultivation for seed multiplication was done in the fields in IRRI, Los Baños (14° 11” N, 121° 15” E) in the Philippines. Mature seeds of sorghum BTx623-Rooney treated with 0.28% EMS to generate a mutant population. The M_1_ seeds were grown in 250 ml plastic cups filled with soil fertilized with 30 kg per hectare of nitrogen, phosphorus and potassium (NPK) combined at a ratio of 3:1:1. The cups were laid out in 1 m^2^ plot(s). Three weeks old seedlings were transplanted into soil with 10 cm x 75 cm plant spacing. Seedlings were fertilized with a basal dose of NPK at the rate of 30 kg ha^-1^. Urea was added at a rate of 97 kg ha^-1^ at 21 and 35 days after transplantation (DAT). A sprinkler irrigation system was used. Around 100 DAT, the M_2_ seeds were harvested, air-dried for four days at 40°C to bring down the moisture content between 8 and 10%. Each panicle was treated as a line. The seeds were stored in a 4°C cold room. For the cultivation of M_2_ generation, we used lines that produced more than 50 seeds. From a population of one million M_1_ seeds, 35,000 individual panicles were advanced to the M_2_ generation.

More than 35,000 M_2_ lines were sown. For each M_2_ line, 24 seeds were grown. Each seed was sown in seedling trays (Ronaash^®^ Slim, Rannoch) containing 50 cells of 100 ml capacity. Each cell was filled with fertilized soil (0.025 g NPK kg^-1^ soil combined at a ratio of 3:1:1). The seedling trays were grown in field conditions. Seven days after sowing (DAS) percentage germination and visual phenotypes were recorded and seedlings with the desired phenotypes were selected. The selected plants were transplanted into 8 L pots and fertilized with 0.025 g NPK kg^-1^ soil combined at a ratio of 3:1:1. Pots were irrigated daily. Around 100 DAT, the M_3_ seeds were harvested, air-dried for four days at 40°C and stored at 4°C cold room until used.

### Phenotyping

A week after sowing, the number of seeds that germinated, the frequency of visual phenotypes such as albino, pale, crinkly and slow growing plants were recorded to analyze the occurrence of such mutants. Disadvantaged (pale, crinkly and slow growing) mutants were sampled for stable ^13^C isotope analysis. The rate of photosynthesis was also measured for some pale and slow growing mutants using LI-6400 portable photosynthesis systems. After the measurements and analysis, the seedlings were grown in a high CO_2_ chamber at 10,000 ppm for rescue and 25 ± 2°C for 12 hours during the night and 29 ± 2°C during the day.

### Dry matter δ^13^C analysis

After measurement of gas exchange, sections of the same leaf were sampled for dry matter and microscopic analysis. Tissue from the center of the leaf blade was harvested, dried overnight in an oven at 80°C, and ground to a fine powder. Dry matter carbon isotope composition measurements were performed as previously described [39]. One to two mg samples were flash-combusted in a CE1110 CHN-S analyzer (Carlo Erba, UK) and the CO_2_ isotopic composition determined by mass spectrometry in a Fisons Isochrom CF-IRMS (Continuous-flow Isotope Ratio Mass Spectrometer; Isoprime, UK). The δ^13^C signatures are presented as isotopic ratios (per mill, ‰) relative to the isotopic standard Pee Dee Belemnite.

### Gas exchange measurements

Plants selected for gas exchange measurement were watered in the morning and kept in the area of measurement for at least 90 minutes for acclimatization of the plant prior to the actual measurement. The third and the fourth leaves were clipped by the head of the infrared gas analyzer (IRGA) of the LI-6400XT portable photosynthesis system (LICOR Biosciences, Lincoln, NE, USA). The leaf temperature was maintained at 30°C as per the temperature of the measurement room, with a light intensity of 2,000 μmol m^-2^ s^-^. The mean atmospheric pressure at the measurement site (38 m above the sea level) was 94.8 kPa. A constant airflow of 400 μmols^-1^ was maintained and the leaf-to-air vapor pressure deficit was between 1.0 and 1.5 kPa. The leaves were acclimated in the cuvette for about 30 minutes.

The response curves of the rate of CO_2_ assimilation (*A*, μmolCO_2_ m^-2^s^-1^) to the changes in intercellular CO_2_ concentration (*Ci*, μmolCO_2_mol^-1^) were acquired by increasing CO_2_ concentration in the cuvette (*Ca*) from 0 to 2000 μmol CO_2_ mol^-1^ at a photosynthetic photon flux density (PPFD) of 2000 μmol m^-2^s^-1^. The carboxylation efficiency (CE) was calculated from the initial slope (*Ci* < 100) of the *A-Ci* curves; and CO_2_ compensation point (CP, μmolCO_2_ per mol_air_) was taken from the point of intercept between the *A*-*Ci* curve and the X-axis [40].

Similarly, the light-response curves were obtained by plotting photosynthetic rate against PPFD from 2000 to 0 μmol_quanta_.m^-2^s^-1^ at a constant *Ca* of 400 μmolCO_2_mol^-1^_air_. The quantum efficiency (QE) was calculated from the initial slope of the light-response curves using the first four points of PPFD that were less than 100 μmol photons m^-2^ s^-1^[41]. The light compensation point was taken from the point of intercept between the light response curve and the X-axis. All other conditions were as in the ACi measurement.

### Chlorophyll fluorescence microscope imaging

The third leaf, when it was the youngest fully expanded leaf of the seedling, was sampled. Three cm long leaf strips from the middle part of the leaf blade were cut, fixed in 2.5% glutaraldehyde, placed in scintillation vials, vacuum infiltrated (20 psi) for 20 minutes and stored at 4°C overnight. Using a sharp razor blade, very fine sections of leaf were cut and stained with 0.05% calcofluor-white. Leaf sections were mounted in glycerol, viewed and imaged using a fluorescence microscope (Olympus DSU (Disc Spinning Unit) Confocal System, Japan). The total chlorophyll content or “greenness” was measured using Chlorophyll meter SPAD. The data from the SPAD reading are comparable to the greenness of the leaves [42].

### Stomatal density

For each plant type (low δ^13^C, normal δ^13^C, and WT) four representative plants were sampled. For each sample, the stomatal densities in 10 sections were recorded and their mean and standard deviation calculated. A section of the third leaf that was sampled for chlorophyll fluorescence imaging was used to study the density of stomata. The abaxial and adaxial epidermis of the leaf were gently cleaned using a damped paper towel, carefully smeared with nail varnish in the mid-area between the central vein and the leaf edge and left for 20 min until dry. The thin film (approximately 5 mm×10 mm) was peeled off the leaf surface, mounted on a glass slide, and viewed using a light microscope (Olympus BX63, Japan).

### Crossing and advancement of generation

The sorghum mutant was crossed to its WT (BTx623-Rooney) to obtain both cross- and self-pollinated seeds following a segregation crossing strategy [43]. The BC_1_F_2_ was used for segregation analysis of *δ*^13^C and for whole genome re-sequencing.

### Leaf sampling, DNA extraction, quality control, and sequencing

Young and tender leaves were transferred into liquid nitrogen. One leaf each from five seedlings were pooled and then ground to a fine powder for DNA isolation. Total DNA was extracted following the CTAB method [44] and checked for quality by running on a 1.5% agarose gel. The concentration of DNA was measured using a nanodrop (ND-8000, Thermo Scientific). Equal concentrations of DNA from six individual sub-pools were pooled to obtain 25μg DNA. Four pools were prepared namely SbPool 1 to SbPool 4, such that each pool had equal amount of DNA from 30 seedlings. The SbPool 1 and SbPool 2 contained DNA from samples of slow growing pale mutant seedlings with low δ^13^C. The SbPool 3 DNA was obtained from small plants with normal δ^13^C, and the SbPool 4 DNA was obtained from normal BC_1_F_2_ seedlings with normal δ^13^C (S1 Fig). The DNA pools were sequenced using HiSeq2500 PE125 sequencing strategy (BGI Tech Solutions Co., Shenzhen, China). The WT samples were also sequenced for comparison of sequences against the mutants. The raw read data for this project have been submitted to the Sequence Read Archive (SRA) of the National Center for Biotechnology Information (NCBI) under BioProject ID PRJNA384699. The SRA accession numbers are SRX2768409, SRX2771011, SRX2771012 and SRX2771013. The sequence data of wildtype sorghum was submitted previously with SRA accession number SRX973468.

### Construction of individual genome for wildtype (BTx623-Rooney)

An alignment of WT reads onto Sorghum reference genome (BTx623) showed the WT sequences varied from the reference genome in a number of positions. Therefore, construction of an individual genome for the WT [12] was preferred and submitted to SRA accession number SRX973468. In short, the steps involved filtering reads for base quality using FASTX toolkit (parameters used: base quality ≥ 20, minimum length after 3' trimming ≥ 30, and fraction of read length with high-quality bases ≥ 0.85), followed by their alignment with default parameters except that only paired-end reads were used for alignment. Reads of size ~100 bp were aligned using BWA-MEM v0.6.9 (http://bio-bwa.sourceforge.net/) and that ~50 bp were aligned using Bowtie2 [45]. The WT genome was reconstructed from the alignment by Pilon [46], a tool used for assembly improvement. To further improve the quality of the individual WT genome, Pilon was run iteratively eight times so that the detected variants stabilized.

### Processing of short sequence reads and variant calling

The quality of sequencing data was initially evaluated using FastQC (http://www.bioinformatics.babraham.ac.uk/projects/fastqc/), followed by read trimming/ filtering for base or read quality using Trimmomatic v0.32; [47]. The parameters used were leading = 10, trailing = 10, sliding window = 5:15, and minimum length = 50. The filtered reads were aligned to BTx623-Rooney genome by BWA-MEM (v0.7; http://bio-bwa.sourceforge.net/) with default parameters. The alignment was improved by (1) fixing mate information by samtoolsv1.2 [48], (2) realignment around InDels, (3) base quality re-calibration using a set of very high confidence variants, and (4) mark PCR duplicates by Picard v1.96 (http://broadinstitute.github.io/picard/). The steps 2 and 3 were carried out using GATK v3.3–0 [49]. Variant calling was done for individual samples using HaplotypeCaller, a part of GATK followed by joint genotyping of all four samples using GenotypeGVCF, a part of GATK. The variants were filtered using in-house PERL scripts for (1) those not induced by EMS (other than G->A or C->T), although other types of SNPs may be induced; but it was not considered in this analysis mainly to improve the quality of SNP discovery (2) read depth outside the range of 10–150, (3) allele frequency less than 0.3, (4) genotype quality less than 30, and (5) any WT allele. Despite these stringent filters, few loci in the genome had usually high density of variants, mainly arising from the repetitive regions. Such variants were filtered (only from that region of chromosome 10 where the association was observed) by masking the repetitive regions. The sequence fragments in hard masked BTx623 reference genome were extracted and mapped back to BTx623-Rooney genome. The variants in the mapped region were used for further analysis.

#### Causal gene discovery

For the discovery of causal gene, the region of the genome linked to the phenotype in all mutant lines in the pool was searched as described in MutMap [15]. For that, the alternate allele frequency (AF), which is the ratio of the number of reads supporting the mutant allele to the total number of reads aligned, was plotted for each of the ten chromosomes. The SNPs from the region of the genome with AF = 1 in mutant pools were extracted (to avoid any putative causal SNP to be missed out, the filters described previously for SNP calling were relaxed for SNPs of this region and each case was examined manually) and were annotated using in-house Perl scripts to find out if any of them caused amino acid change or introduced a stop codon. The genes with either of the two changes were chosen as candidate genes and were subjected to further tests to find the causal gene. The information on the nine candidate genes was collected from the databases such as Phytozome 10.3, Gramene, Quick GO etc., and published literature on sorghum or other species [50, 51].

### Leaf sampling, RNA extraction, and quality control

The fully expanded third leaf was sampled from 10:00 to 10:30 am and immediately frozen in liquid nitrogen. The leaf was ground in liquid nitrogen and total RNA was extracted using TRIZOL reagent following the manufacturer’s instructions (Invitrogen, USA). RNA integrity was checked by running 1 μg of RNA on a 2% agarose gel in 1X TAE running buffer. Total RNA was treated with RQ1 RNAse free DNAse (Promega, USA) followed by phenol-chloroform purification. One microgram of the purified RNA was used as the template to synthesize cDNA through reverse transcription using a first strand cDNA synthesis kit (Roche Diagnostics, Germany). The concentration of cDNA was normalized to 100 ng/μl and used for PCR amplification using the primers specific to the candidate genes.

### Gene expression analysis by semi-quantitative PCR

Semi-quantitative PCR with SYBR Green I Master mix (Roche Diagnostics, Germany) in a final reaction volume of 20 μl was performed with primers specific to the gene of interest (S1 Table). The EF1α was used as an internal control. Relative transcript abundance quantification was quantified based on band intensity using the Image J [https://imagej.nih.gov/ij/].

## Supporting information

S1 Fig A plot of alternate allele frequency (AF) in mutant pool along all chromosomes. The X-axis shows the position in Mb units in respective chromosome whereas Y-axis shows the AF values.
(TIF)

S2 Fig A focus on the target region of alternate allele frequency (AF) plot in theq arm of chromosome ten of the mutant. The X-axis shows the position in Mb units in respective chromosome whereas Y-axis shows the AF values.
(TIF)

S3 Fig An expression profile of Sobic.010G266000 an ortholog related with chloroplast based on the maize atlas [50]. (TIF)

S4 Fig Profile of expression of Sobic.010G250100 in maize primordial tissues [51] based on maize atlas. Tissue name: FP = foliar primordial plastochron 1; FP34 = foliar primordial plastochron 3 or 4; FP5 = foliar primordial plastochron 5; FI = foliar immature; FE = foliar expanded.
(TIF)

S5 Fig The expression profile of all the candidate genes based on maize atlas. (TIF)

S6 Fig Semi-quantitative PCR analysis of candidate genes of mutant 33 BC_1_F_2_ seedlings. The Sobic.010G239700, Sobic.010G249000, Sobic.010G250100, Sobic.010G264000, and Sobic.010G241900 showed no apparent changes in the transcript expression compared to the wild type. EF1α is the housekeeping gene.
(TIF)

S1 Table List of SNPs from a region of chromosome 10 with AF = 1 in the mutant pool with low δ^13^C (SbPool 1) whereas AF< = 0.6 in WT pool with normal δ^13^C (SbPool 4). The annotations of the genes are according to phytozome v.02.
(XLSX)

S2 Table The detail of 37 SNPs in the region with high alternate frequency. (XLSX)

S3 Table List of primers specific to the gene of interest. (XLSX)
