# Supplementary figures and images for: A sorghum (Sorghum bicolor) mutant with altered carbon isotope ratio
Source: PLoS One. 2017 Jun 22;12(6):e0179567. doi: 10.1371/journal.pone.0179567 (PMC5480886; doi:10.1371/journal.pone.0179567)

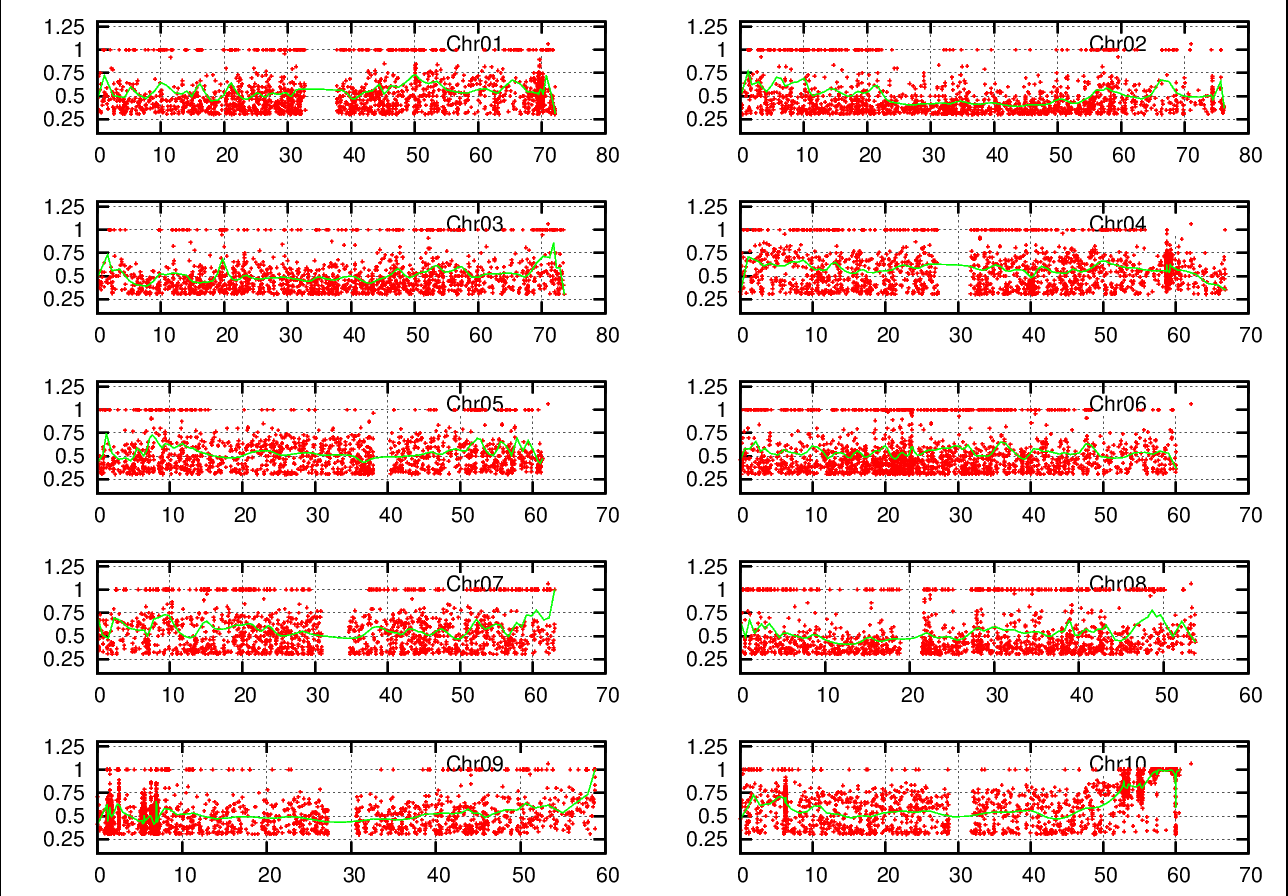

Supplement: S1 Fig — The X-axis shows the position in Mb units in respective chromosome whereas Y-axis shows the AF values. (TIF) [file pone.0179567.s001.tif]

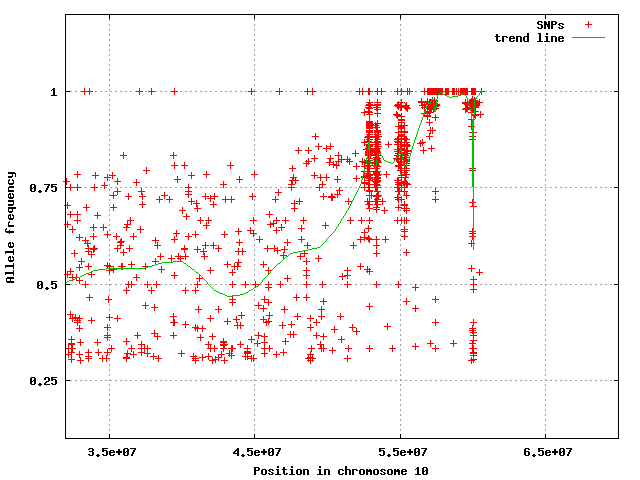

Supplement: S2 Fig — The X-axis shows the position in Mb units in respective chromosome whereas Y-axis shows the AF values. (TIF) [file pone.0179567.s002.tif]

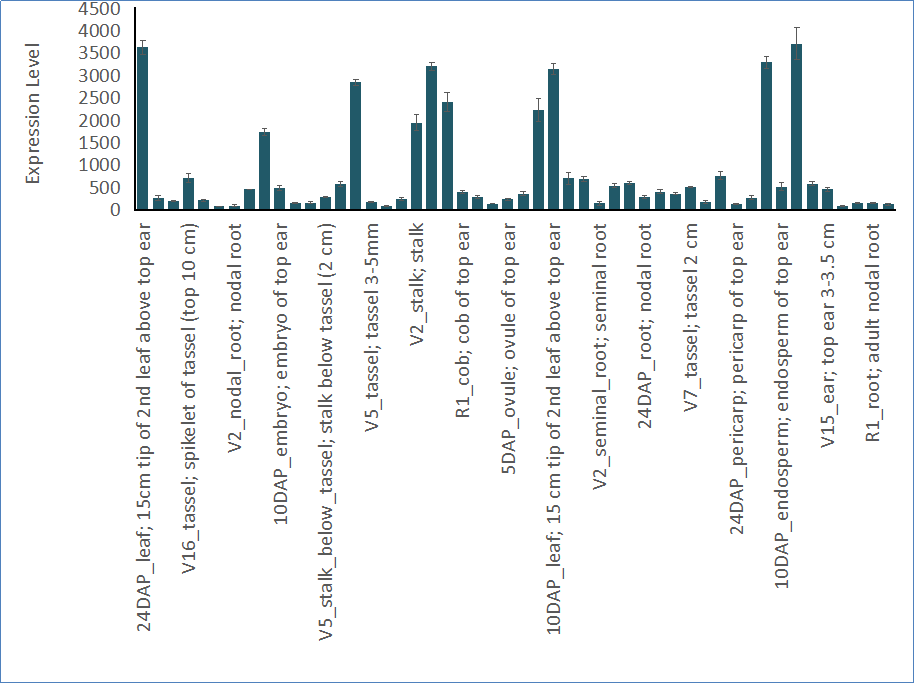

Supplement: S3 Fig — (TIF) [file pone.0179567.s003.tif]

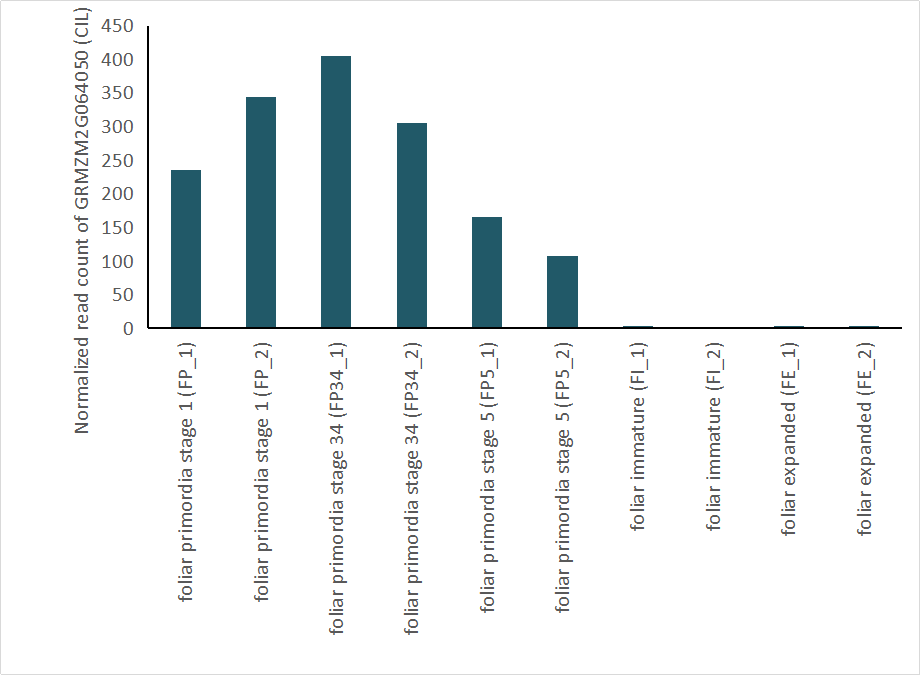

Supplement: S4 Fig — Tissue name: FP = foliar primordial plastochron 1; FP34 = foliar primordial plastochron 3 or 4; FP5 = foliar primordial plastochron 5; FI = foliar immature; FE = foliar expanded. (TIF) [file pone.0179567.s004.tif]

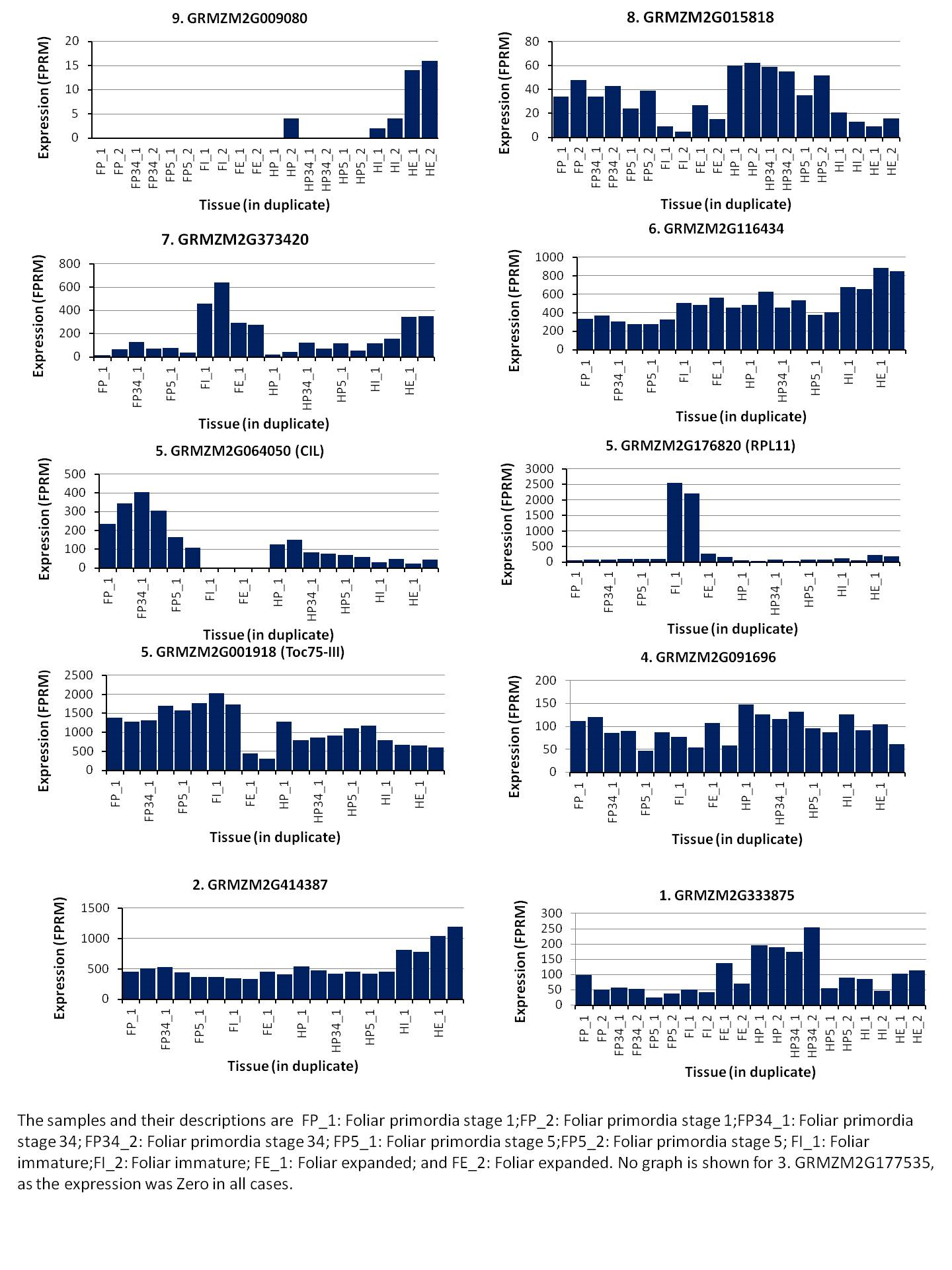

Supplement: S5 Fig — (TIF) [file pone.0179567.s005.tif]

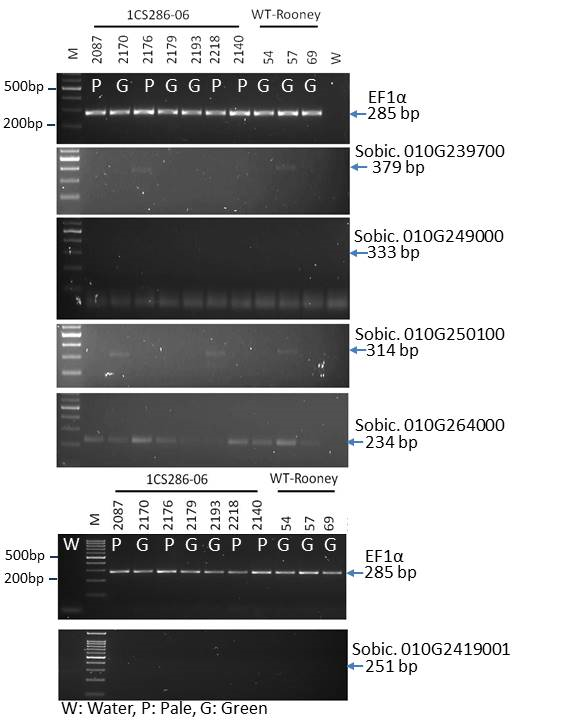

Supplement: S6 Fig — The Sobic.010G239700, Sobic.010G249000, Sobic.010G250100, Sobic.010G264000, and Sobic.010G241900 showed no apparent changes in the transcript expression compared to the wild type. EF1α is the housekeeping gene. (TIF) [file pone.0179567.s006.tif]
